# Utility of next-generation sequencing in identifying congenital erythrocytosis in patients with idiopathic erythrocytosis

**DOI:** 10.3389/fmed.2024.1440712

**Published:** 2024-09-06

**Authors:** Saša Anžej Doma, Nika Kraljić, Aleša Kristan, Nataša Debeljak, Aleš Maver, Tadej Pajič, Irena Preložnik Zupan

**Affiliations:** ^1^Hematology Department, University Medical Centre Ljubljana, Ljubljana, Slovenia; ^2^Department of Internal Medicine, Faculty of Medicine, University of Ljubljana, Ljubljana, Slovenia; ^3^Institute of Biochemistry and Molecular Genetics, Faculty of Medicine, University of Ljubljana, Ljubljana, Slovenia; ^4^Clinical Institute of Genomic Medicine, University Medical Centre Ljubljana, Ljubljana, Slovenia; ^5^Clinical Biochemistry, Faculty of Medicine, University of Maribor, Maribor, Slovenia

**Keywords:** non-clonal erythrocytosis, congenital erythrocytosis, next-generation sequencing, erythropoietin, hemochromatosis

## Abstract

**Background:**

Congenital erythrocytosis (CE) is increasingly recognized as the cause of erythrocytosis in patients in whom polycythemia vera and secondary acquired causes have been excluded. The aim of our study was to determine possible genetic background in patients with idiopathic erythrocytosis.

**Methods:**

40 patients with idiopathic erythrocytosis, referred to our institution in a 5-year period, were analyzed. We collected data on erythropoietin (Epo) levels, hemoglobin (Hgb), hematocrit (Hct), erythrocyte count, age, gender, past thrombotic events, concomitant diseases, and smoking status. CE was tested using next-generation sequencing (NGS), in the majority of patients also measurement of P50 and Hgb electrophoresis were performed. Patients with signs of iron overload were tested for genetic variants in the *HFE* gene.

**Results:**

The median patient age at analysis was 46.5 years (range 22–73), with 37 out of 40 being males (93 %). The median Hgb, Hct and red blood cells count were 180 g/L, 0.51, 5.985 x 10^12^/L in men and 171 g/L, 0.50 and 5.68 x 10^12^/L in women, respectively. Epo levels were decreased in three, increased in one patient and within the normal range in the rest (median 7.55 mIU/mL; range 2.90–19.50). Eight patients (20 %) smoked. 32 (80 %) were treated with low-dose aspirin, and 20 (50 %) underwent at least one phlebotomy. Thromboembolic events were recorded in 2 patients (5 %). P50 was measured in 20 out of 40 patients, and it was above 24 mm Hg (3.12 kPa) in all of them. Hemoglobin electrophoresis was performed in 73 % of patients, with no abnormal Hgb detected. Variants in the *HFE* gene were found in 8 out of 40 patients (20 %), but in only one patient the results were associated with an increased risk for hemochromatosis. Although no pathogenic variants for CE were detected by NGS, two variants of uncertain significance, namely *EGLN1* (NM_022051.2):c.1072C>T (p.(Pro358Ser)) and *EGLN1* (NM_022051.2):c.1124A>G (p.(Glu375Gly)) were identified as strong etiologic candidates.

**Conclusion:**

CE is an extremely rare condition. Genetic testing is advised in young individuals with a long-standing persistent erythrocytosis, possibly with a family history and after exclusion of more frequent secondary causes and polycytemia vera.

## 1 Introduction

Idiopathic erythrocytosis is an ill-defined terminology that presumes the existence of an increased hemoglobin/hematocrit (Hgb/Hct) level without an identifiable etiology. JAK2 unmutated or non-polycythemia vera (PV) erythrocytosis has a higher prevalence than PV and it represents a spectrum of heterogeneous diseases ranging from hereditary to acquired medical conditions. Acquired erythrocytosis is most frequently a compensatory mechanism in response to a hypoxic condition such as cardiopulmonary disease, hypoventilation (sleep apnea, snoring…), living at high altitudes, tobacco use, but can also be a result of certain medications (diuretics, sodium-glucose cotransporter 2 inhibitors, anabolic steroids) or supplements (androgens), and erythropoietin (Epo). Ectopic secretion of Epo by renal or hepatic tumors, renal artery stenosis etc is another mechanism of secondary erythrocytosis (1).

Although rare, congenital erythrocytosis (CE) is increasingly recognized as the cause in many patients in whom acquired, especially neoplastic causes have been excluded. During the past two decades, the underlying molecular mechanisms of CE are increasingly getting unraveled. Causes of CE include pathogenic variants in the Epo receptor gene (*EPOR*), components of the oxygen-sensing pathway such as the alpha subunit of the hypoxia-inducible transcription factor (*EPAS1*, also known as *HIF2A*), prolyl hydroxylase Egl nine homolog 1 (*EGLN1*, also known as *PHD2*), and the von Hippel-Lindau disease tumor supressor (*VHL*). Additionally, variants in globin genes leading to formation of high oxygen affinity hemoglobins (*HBA, HBB*) and rarely, bisphosphoglycerate mutase (*BPGM*) variants, affecting the 2,3-bisphosphoglycerate levels, can also increase the oxygen affinity of Hgb and cause CE (2).

**FIGURE 1:**
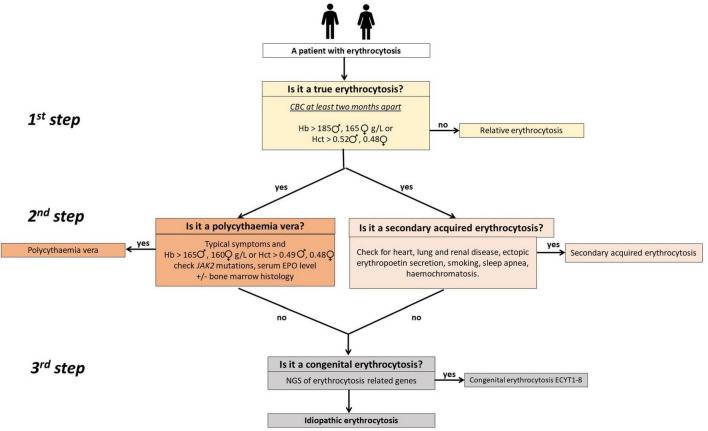
A simple three-step clinical algorithm for diagnosing patients with erythrocytosis (3). *The first step* involves at least two complete blood count (CBC) measurements 2 months apart to confirm true erythrocytosis. Use of the red cells mass measurement can obviate it. In *the second step*, patients with polycythemia vera are identified or ruled out through testing for JAK2 mutations (V617F and exon 12 when applicable) and assessing serum EPO levels. Bone marrow histology may be necessary for some patients. Concurrently, secondary acquired erythrocytosis is assessed by examining patient history, clinical and laboratory data, and results from other diagnostic procedures. In cases where increased iron stores are detected, it is reasonable to check for common mutations in iron homeostatic regulatory genes (HFE), such as c.845G>A (p.C282Y), c.193A>T (p.S65C), and c.187C>G (p.H63D). In *the third step*, patients with suspected congenital erythrocytosis are referred for genetic testing using targeted next-generation sequencing (NGS). Adapted from (3).

We have previously developed a diagnostic algorithm to improve diagnosis and management of patients with non-clonal erythrocytosis (3). It is depicted in Figure 1. The aim of this prospective study was to determine possible genetic background using next-generation sequencing (NGS) in carefully selected patients with idiopathic erythrocytosis, following exclusion of all other causes.

## 2 Materials and methods

### 2.1 Patients

We prospectively collected patients referred to our hematology department between 2019 and 2024, in whom PV and secondary causes of erythrocytosis were excluded using the aforementioned algorithm (3).

Patients were included if they had unexplained non-clonal persistent erythrocytosis (Hct above 0.52 and 0.48 and/or Hgb above 185 g/L and 165 g/L in men and women, respectively). Additionally, some patients with lower values were referred for genetic testing if there was a strong suspicion of a congenital origin (low Epo, thrombotic complications, family history) or if they were already undergoing regular venesections. The exclusion criterion was obvious secondary erythrocytosis. However, some smokers were included due to either inappropriately low Epo level and/or unusually high Hct or Hgb level, considering the possibility that patients who smoke may still have underlying secondary erythrocytosis.

In patients with subnormal Epo bone marrow biopsy was performed, but in none of the patients myeloproliferative neoplasm was confirmed. In some of the patients, in whom myeloproliferative disease was suspected initially, also *CALR* and *MPL* were tested, although they are rarely associated with PV (1). All patients with subnormal Epo underwent testing for *JAK2* exon 12 variants.

Secondary erythrocytosis had been excluded before by investigating the patients’ comorbidities, such as lung, heart, and renal diseases, obesity, obstructive sleep apnea, and Epo-secreting tumors. This was done using imaging techniques, pulse oximetry, and blood gas analysis, including the measurement of carboxyhemoglobin (CO-Hb) and polysomnography. Data on smoking and concomitant drug use were also collected.

Demographic data, including age, gender, concomitant diseases and hematologic parameters (Hgb, Htc, erytrocyte count) were collected. All available patients’ values were included in the calculation of median values. Serum Epo levels were determined by Solid Phase Sandwich ELISA test (R&D Systems, Inc., Abingdon, UK). Hgb electrophoresis and oxygen pressure at 50 % Hgb saturation (p50) was performed from venous blood; values of p50 below 24 mm Hg were considered pathological. In patients who demonstrated increased iron stores, the most common variants linked to hemochromatosis in the homeostatic iron regulatory gene (*HFE)*, namely c.845G>A (C282Y), c.193A>T (S65C), and c.187C>G (H63D), were determined by allele discrimination assays (4).

### 2.2 Genetic analysis

For genetic analysis, targeted NGS for erythrocytosis was performed. The genomic DNA of individuals was extracted using QIAamp DNA mini kit (Qiagen) from either isolated cells from peripheral blood (granulocytes or leukocytes) or directly from the whole blood. The commercial reference DNA control NA12878 (Coriell Institute) was also included as a control for sequencing efficiency. Library preparation and enrichment were performed with DNA Prep with Enrichment reagent kit (Illumina) as described previously (5, 6). Custom gene panel for enrichment included 28 genes, that were either known erythrocytosis-causative genes (*EPOR, VHL, EGLN1, EPAS1, EPO, HBB, HBA1, HBA2, BPGM, SH2B3, JAK2*) or new candidates involved in erythrocyte development (*BHLHE4, EGLN2, EGLN3, GFI1B, HIF1A, HIF1AN, HIF3A, KDM6A, OS9, PKLR, GATA1, TET2, PIEZO1, SLC30A10, HFE, SEC23B, ARNT*). Enriched libraries were sequenced on MiniSeq sequencer (Illumina). Genetic variants were classified in accordance with The American College of Medical Genetics and Genomics (ACMG)/The Association for Molecular Pathology (AMP) standards and guidelines for interpretation of sequence variants (7), modified in accordance with Association for Clinical Genomic Science (ACGS) Best Practice Guidelines for Variant Classification in Rare Disease 2024 recommendations (8).

### 2.3 Statistical analysis

The statistical analysis was performed using Statistica 8.0 (StatSoft, Inc. Tulsa, OK, USA) software. The distribution of continuous variables was skewed and, thus, presented as medians with ranges. Categorical data were reported as frequencies and percentages. The differences between the compared groups were analyzed using the Mann–Whitney test. A *p*-value of 0.05 or less was considered statistically significant.

All included patients signed informed consent for inclusion into the study. The study was approved by the National Medical Ethics Committee, Ministry of Health of the Republic of Slovenia, approval no. 115/07/15 (0120-198/2015/4, 0120-287/2019/4, 0120-287/2019/14).

## 3 Results

### 3.1 Clinical and laboratory characteristics of the participants

We included 40 patients with idiopathic erythrocytosis. Their clinical characteristics are demonstrated in Table 1.

**TABLE 1:** Clinical characteristics of patients.

Age (years; median, range)	46.5 (22–73)
Males (number, %)	37 (92.5)
Thromboembolic events (number, %)	2 (5)
Erythropoietin level (IU/L; median, range)	7.55 (2.90–19.50)
Hemoglobin (g/L; median, range)	179 (158–197)
Haematocrit (/; median; range)	0.51 (0.47–0.58)
Red blood cell count (10^12^/L; median, range)	5.98 (5.38–7.75)
Platelet count (10^9^/L; median, range)	223 (143–363)
White blood cell count (10^9^/L; median, range)	7.61 (3.53–18.1)
Ferritin concentration (μg/l; median, range)	114 (13–592)
Transferrin saturation (%; median, range)	41 (19–71)

8/40 (20 %) of the patients were smokers. The most frequent accompanying diseases were arterial hypertension, hyperlipidemia and asthma (5/38). There were three patients with subnormal Epo levels and one patient with elevated Epo, with normal values ranging between 3.3 and 16.6 IU/L. In the majority of patients Epo was in the normal range. The median Hgb, Hct and red blood cell count were 180 g/L, 0.51, 5.985 x 10^12^/L in men and 171 g/L, 0.50 and 5.68 x 10^12^/L in women, respectively.

The *JAK2* V617F variant was an exclusion criterion, so all the patients were tested for it before the study. Additionally, we tested 32/40 (80 %) patients for *JAK2* exon 12, 17 (43 %) for *CALR*, 16 (40 %) for *MPL* W515L/K and 11 (28 %) patients for *BCR::ABL1*; all patients were negative for the variants as well. Bone marrow aspirates were performed on 19 (48 %) patients, in none of them the findings were indicative of myeloproliferative disease. 32/40 (80 %) patients were treated with low dose aspirin (100 mg daily) and 20/40 (50 %) of patients had at least one phlebotomy.

Hemochromatosis was tested in 15 (38 %) patients, among whom, only 3 patients demonstrated signs of iron overload according to the latest criteria for hemochromatosis (9). In only one patient a compound heterozygous genotype (C282Y/S65C) for *HFE* was detected, which is associated with low increased risk for hemochromatosis (10–12). In 7 patients heterozygous variants in the *HFE* gene were detected, which are not known to be associated with increased risk of hemochromatosis, namely H63D in 3 patients, C282Y in 3 patients and in S65C in 1 patient. All the patients with identified variants in *HFE* gene were male and had significantly higher levels of ferritin and transferrin saturation, compared to the others. There were no significant differences in other parameters (Hgb, Hct, red blood cell count, Epo levels) between the two groups (Table 2).

**TABLE 2:** Laboratory characteristics of patients, in whom mutations in *HFE* genes were detected (*n* = 8).

Ferritin concentration (μg/l, median, range)	291 (79–447)
Transferrin saturation (%; median, range)	53 (37–71)
Hemoglobin (g/L; median, range)	181 (165–190)
Hematocrit (%; median, range)	0.51 (0.50–0.54)
Red blood cell count (10^12^/L; median, range)	5.92 (5.73–6.31)
Erythropoietin level (IU/L; median, range)	6.13 (3.2–11.7)

Hgb electrophoresis was performed on 29 (73 %) of patients, but no abnormal hemoglobins were detected. P50 was measured in 20/40 patients and was above 24 mm Hg (3.12 kPa) in all of them (median 3.66 kPA; range 3.20–3.88).

### 3.2 NGS analysis

The NGS analysis did not detect any of the known genetic variants, associated with CE. However, two missense variants of uncertain significance (VUS) were identified in *EGLN1* and are presented in Table 3. The first variant was c.1124A>G (NM_022051.2), identified in one patient in a heterozygous state. This variant causes a substitution of amino acid glutamic acid with glycine at position 375 in the aminoacid sequence coded by gene *EGLN1* (p.(Glu375Gly)). The second variant was c.1072C>T (NM_022051.2) that led to an amino acid change at position 358 in the aminoacid sequence EGLN1 p.(Pro358Ser) and was present in a heterozygous state in 2 brothers.

**TABLE 3:** Rare variants identified in patients with targeted NGS for erythrocytosis.

Genomic location (assembly UCSC, hg19)	Gene	Coding DNA change (RefSeq transcript)	Protein change	RS number	Allele frequency (GnomAD)	Genotype	N of cases	Classification of variant* (evidence categories)
*chr1-231506332-T-C*	*EGLN1*	c.1124A>G (NM_022051.2)	p.(Glu375Gly)	NA	NA	Het	1	VUS (PM1_SUP, PM2, PP3, PP4)
*chr1-231506384-G-A*	*EGLN1*	c.1072C>T (NM_022051.2)	p.(Pro358Ser)	NA	NA	Het	2 (brothers)	VUS (PM2, PP3, PP4)

*Classification of variants based on The American College of Medical Genetics and Genomics (ACMG)/The Association for Molecular Pathology (AMP) standards and guidelines for interpretation of sequence variants (7), modified in accordance with Association for Clinical Genomic Science (ACGS) Best Practice Guidelines for Variant Classification in Rare Disease 2024 recommendations (8). Het, heterozygous; NA, not available; RS, reference SNP; UCSC, University of California Santa Cruz; VUS, variant of uncertain significance.

## 4 Discussion

Patients included into the study all presented with consistently elevated Hgb, Hct or erythrocyte count without an obvious reason, however not all of them met true criteria for absolute erythrocytosis. To demonstrate that Hgb levels exceed the norm, it’s imperative to determine the red cell mass (13), which is not possible at our centre and also in many others (14). Of note, as the threshold for PV exclusion has lowered with the latest WHO 2016 classification to Hct of 0.49 in men and 0.48 in women (15), the number of patients with unexplained erythrocytosis increased. With the aim of not missing a potential CE, especially as some types are not associated with extremely elevated erythrocyte count, we decided to include also some younger patients with less pronounced idiopathic erythrocytosis, in whom CE was likely. It is not surprising that the majority (92 %) of patients were men, as the current Hgb/Hct threshold for PV is nearly within the normal range for men. Consequently, men are more frequently referred for PV exclusion compared to women, who rarely exceed the cut-off values. This finding has also been reported by other researchers (16, 17). It is frequently observed also that erythrocytosis resolves during the follow-up, which can be explained by a temporary trigger [smoking of a water-pipe can result in considerable erythrocytosis (18)] and/or intervention, such as venepunction. Additionally, recently approved drugs, such as SGLT-2 inhibitor therapy, are less known triggers of erythrocytosis (1). This is the reason why the median Hct, Hgb and erythrocyte count in our (male) patients were below the inclusion criteria, as all the available values of the patient were included in the calculation. Notably, all of our patients had isolated erythrocytosis, with platelets and white blood cells in the normal range, which is usually not the case in PV patients (15).

When suspecting CE, we can differentiate them based on Epo levels. A subnormal serum Epo is suspicious for presence of an *EPOR* variant. In a normal or elevated Epo, high oxygen affinity Hgb variants are most likely (*HBA, HBB*). A left shift of the oxygen dissociation curve or when venous P50 < 24 mm Hg, is a good screening test for high oxygen affinity Hgb variants, but can also indicate 2,3-bisphosphoglycerate deficiency, methemoglobinemia or *PIEZO1* variants. Germline variants in the oxygen sensing pathway (genes *VHL, PHD2*, and *HIF2A*) do not affect the venous P50 measurement and Epo is either elevated or inappropriately normal. At normal P50 we could also expect germline variants in the *EPO* gene (1). Half of our patients had P50 measured and none of them had a pathological result. The majority of our patients also underwent Hgb electrophoresis, an alternative method for detecting high oxygen affinity hemoglobin variants (1). Also using this method, no pathological results were found.

None of the patients included in the study harbored definitely pathogenic variant in the investigated erythrocytosis-associated genes. The fact that CE was not detected in any of the screened patients is not surprising, as it is known that a substantial proportion of patients with idiopathic erythrocytosis remain unexplained (19, 20). Among our cohort of patients, only a minority reported a possible family history, often being uncertain if a family member had erythrocytosis or unable to verify it.

However, genetic analysis revealed two heterozygous missense variants of uncertain significance in *EGLN1* gene. Gene *EGLN1* encodes for the enzyme prolyl hydroxylase, which plays a crucial role in responding to oxygen tension in the body. Under normal oxygen conditions, EGLN1 hydroxylates the hypoxia-inducible transcription factor (HIF). Hydroxylated HIF facilitates binding of VHL, leading to HIF degradation. Under low oxygen conditions, hydroxylase activity is inhibited, resulting in the stabilization of HIF, which induces transcription of *EPO* and regulates erythropoiesis (21, 22). Up to now, 96 different *EGLN1* genetic variants have been reported in patients with erythrocytosis, from whom 36 were classified as pathogenic or likely pathogenic, and in almost all cases, patients were heterozygous (22). Pathogenic monoallelic variants in *EGLN1* represent an established cause of familial erythrocytosis, type 3 (OMIM: 609820) (23). None of the individuals from this study, in whom the *EGLN1* variant was found, exhibited extreme erythrocytosis, all of them had normal Epo levels. This is in line with the literature reporting that patients with *EGLN1* variants develop secondary erythrocytosis with normal or abnormally high Epo levels (13, 19). The first variant, namely c.1124A>G (NM_022051.2), was identified in a man, who has been treated for many years with regular venepunctions due to headaches and aspirin after a transitory ischaemic attack. He also has asthma, which is well controlled and likely not causing erythrocytosis. The other variant, namely c.1072C>T (NM_022051.2) was identified in 2 brothers, in whom only one had pronounced erythrocytosis but was also a smoker. He has been treated with aspirin. None of them suffered any thrombotic complications.

To our knowledge, the first identified variant c.1124A>G (NM_022051.2) in *EGLN1* (p.(Glu375Gly)) has previously not been reported in association with disease in humans. However, the following arguments were found in support of its pathogenic nature: the variant is absent from control populations in GnomAD (24) [PM2] and several amino acid substitutions ((p.(His374Arg), ClinVar ID: 4357 (Accession: VCV000004357.1); p.(Arg371His), ClinVar ID: 4356 (Accession: VCV000004356.1); p.(Pro317Arg), ClinVar ID: 4355 (Accession: VCV000004355.2)) in the vicinity of the identified variant have been reported in patients of familial erythrocytosis suggesting the importance of the identified region for the physiological function of the *EGLN1* protein product (OMIM: 609820) (23, 25–27), [PM1_SUP]. Additionally, algorithms for theoretical pathogenicity prediction uniformly predict the variant as pathogenic [PP3] and the finding was observed in a relatively narrow target of genes, associated with erythrocytoses [PP4]. Based on currently limited available evidence this variant was classified as VUS.

To our knowledge, the second identified variant c.1072C>T in *EGLN1* (p.(Pro358Ser)) has also not been previously reported in patients with a similar clinical presentation. On the same nucleotide position, VUS with a different nucleotide change (c.1072C>A) that causes a substitution of amino acid proline to threonine (p.(Pro358Thr)) was observed in one patient with high Hgb and Hct, according to a recent publication (22). However, the variant is absent from control populations of GnomAD (24) project [PM2], has a predicted damaging effect based on theoretical pathogenicity predictions [PP3], and is in concordance with the specific clinical presentation of the patient [PP4]. Although the identified variant was present in a heterozygous state in both affected family members, the co-segregation evidence was not yet sufficient to upgrade its classification to likely pathogenic and was classified as a VUS.

**FIGURE 2:**
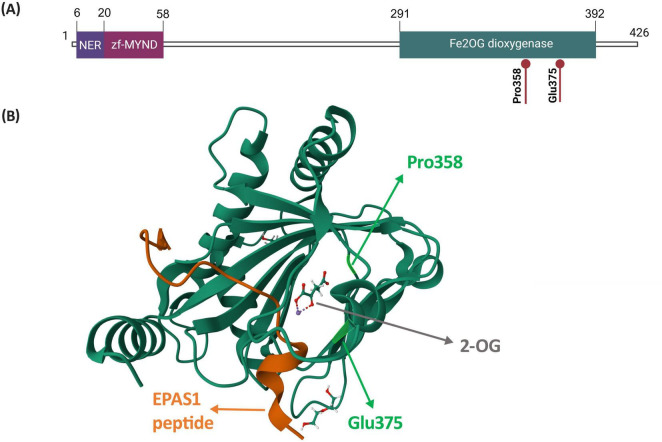
Structure of the EGLN1 protein highlighting amino acids p.Pro358 and p.Glu375. **(A)** Localization of amino acids p.Pro358 and p.Glu375 within a schematic representation of the EGLN1 protein and its domains. Figure was created based on information from Uniprot database (28). **(B)** Localization of amino acids p.Pro358 and p.Glu375 on the EGLN1 structure in complex with 2-oxoglutarate and the EPAS1 peptide, which contains the oxygen-dependent domain. The EGLN1 complex was retrieved from Protein Data Bank (PDB ID: 7Q5X; (29)). Abbreviations: NER, nuclear export region; zf-MYND, MYND-type zinc finger domain; Fe2OG dioxygenase, Fe(II) and 2-oxoglutarate dependent dioxygenase domain, also prolyl hydroxylase domain (PHD); 2-OG, 2-oxoglutarate.

Based on the protein structure, amino acids, on which the two identified variants occur (p.Pro358 and p.Glu375), are both localized in the active site of the EGLN1 hydroxylase (Figure 2). More precisely, the two variants are located in the domain responsible for the hydroxylation reaction (Figure 2A), within the binding pocket of one of the HIF-alpha hydroxylation substrates, 2-oxoglutarate (Figure 2B).

Certain types of CE are associated with thrombotic complications, however, the thrombotic risk of patients with idiopathic erythrocytosis is supposed to be much lower, compared to patients with PV (30). Jalowiec reported that 17 % patients with *JAK2* unmutated polycythaemia patients suffered a thrombosis in a 10-year period, with half of the events occurring in the arterial and the other half in the venous system (16). Two patients from our cohort also experienced thrombosis and we previously reported that thrombotic events were not so rare (12–20%) among patients with non-PV erythrocytosis (3). Frequently, hematologists treat these patients as if they had PV; among our patients 80 % were prescribed aspirin and 50 % had at least one phlebotomy. Gordeuk and colleagues concluded that hematocrit alone may not be sufficient to accurately predict thrombotic risk in patients with idiopathic erythrocytosis. They found that underlying genetic mutations, inflammation, and individual patient characteristics also significantly influence thrombotic risk (31). It is advisable, though, that phlebotomy be used as symptom control in selected cases only and low-dose aspirin for cardiovascular risk optimization. Cytoreductive therapy is contraindicated in non-PV erythrocytosis (30).

As hemochromatosis can rarely manifest with erythrocytosis (1, 32) some patients, particularly those with high iron stores, were tested for haemochromatosis. The association between *HFE* variants (H63D and C282Y) and erythrocytosis has only recently been established. In none of the patients the homozygous C282Y mutation was detected, which is the most typical and frequent genetical finding in hereditary hemochromatosis (9). It has been shown that *HFE* variants are more frequently detected in cases of idiopathic erythrocytosis compared to the general population and seem to promote red cell production. Interestingly, those patients do not present with high ferritin (1, 13, 17, 33). Our results confirm those findings; variants in the *HFE* gene were detected in 8 (20 %) patients, all male, and their median ferritin level did not reach the current diagnostic threshold for haemochromatosis (9). However, three of them underwent at least one phlebotomy. Only one patient was diagnosed with compound heterozigosity, associated with increased risk of hemochromatosis (9, 32). Further investigation of this topic is warranted.

The major limitation of our study is the inability of our center to confirm absolute erythrocytosis, which may result in missing some CE patients or, conversely, incurring excessive costs by testing individuals who do not require it. The identified VUS in *EGLN1* gene require further investigation, such as functional studies, to determine their pathogenicity. Genetic counseling and regular reinterpretation of VUS in probands and/or family members is advised.

## 5 Conclusion

Our study, which did not detect any well-established types of CE, confirms that CE is an extremely rare condition. Of note, variants of uncertain significance in the *EGLN1* gene were detected in 3/40 patients and in the *HFE* gene in 8/40 patients. This is somehow less frequent compared to other studies, but confirms the findings that erythrocytosis appears to be multigenic and often remains unexplained (13, 20, 34). Appropriate management of these conditions is not clearly defined. Therefore, referral for NGS testing should be limited to younger patients with persistent absolute erythrocytosis, particularly those with a family history, after all other possible causes have been excluded. As an expanding array of genetic variants has been identified as potential causes of erythrocytosis over time (2, 13), regular re-evaluation is advised to incorporate new genetic discoveries.

## Data Availability

The raw data supporting the conclusions of this article will be made available by the authors, without undue reservation.
